# Changes in the superior longitudinal fasciculus and anterior thalamic radiation in the left brain are associated with developmental dyscalculia

**DOI:** 10.3389/fnhum.2023.1147352

**Published:** 2023-09-28

**Authors:** Nazife Ayyıldız, Frauke Beyer, Sertaç Üstün, Emre H. Kale, Öykü Mançe Çalışır, Pınar Uran, Özgür Öner, Sinan Olkun, Alfred Anwander, A. Veronica Witte, Arno Villringer, Metehan Çiçek

**Affiliations:** ^1^Department of Neurology, Max Planck Institute for Human Cognitive and Brain Sciences, Leipzig, Germany; ^2^Department of Interdisciplinary Neuroscience, Health Sciences Institute and Brain Research Center, Ankara University, Ankara, Türkiye; ^3^Subproject A1, CRC 1052 “Obesity Mechanisms”, University of Leipzig, Leipzig, Germany; ^4^Department of Physiology, School of Medicine, Ankara University, Ankara, Türkiye; ^5^Neuroscience and Neurotechnology Center of Excellence, Ankara, Türkiye; ^6^Program of Counseling and Guidance, Department of Educational Sciences, Faculty of Educational Sciences, Ankara University, Ankara, Türkiye; ^7^Department of Child and Adolescent Psychiatry, School of Medicine, Izmir Democracy University, Izmir, Türkiye; ^8^Department of Child and Adolescence Psychiatry, School of Medicine, Bahçeşehir University, Istanbul, Türkiye; ^9^Department of Elementary Education, Faculty of Educational Sciences, Ankara University, Ankara, Türkiye; ^10^Department of Neuropsychology, Max Planck Institute for Human Cognitive and Brain Sciences, Leipzig, Germany; ^11^MindBrainBody Institute, Berlin School of Mind and Brain, Charité and Humboldt University, Berlin, Germany

**Keywords:** anterior thalamic radiation, diffusion-weighted magnetic resonance imaging, mathematical learning disability, probabilistic tractography, superior longitudinal fasciculus, arithmetic and numerical abilities, childhood white matter, tract length

## Abstract

Developmental dyscalculia is a neurodevelopmental disorder specific to arithmetic learning even with normal intelligence and age-appropriate education. Difficulties often persist from childhood through adulthood lowering the individual’s quality of life. However, the neural correlates of developmental dyscalculia are poorly understood. This study aimed to identify brain structural connectivity alterations in developmental dyscalculia. All participants were recruited from a large scale, non-referred population sample in a longitudinal design. We studied 10 children with developmental dyscalculia (11.3 ± 0.7 years) and 16 typically developing peers (11.2 ± 0.6 years) using diffusion-weighted magnetic resonance imaging. We assessed white matter microstructure with tract-based spatial statistics in regions-of-interest tracts that had previously been related to math ability in children. Then we used global probabilistic tractography for the first time to measure and compare tract length between developmental dyscalculia and typically developing groups. The high angular resolution diffusion-weighted magnetic resonance imaging and crossing-fiber probabilistic tractography allowed us to evaluate the length of the pathways compared to previous studies. The major findings of our study were reduced white matter coherence and shorter tract length of the left superior longitudinal/arcuate fasciculus and left anterior thalamic radiation in the developmental dyscalculia group. Furthermore, the lower white matter coherence and shorter pathways tended to be associated with the lower math performance. These results from the regional analyses indicate that learning, memory and language-related pathways in the left hemisphere might be related to developmental dyscalculia in children.

## 1. Introduction

Developmental dyscalculia (DD or mathematical learning disability) is a neurodevelopmental disorder that negatively affects learning of numerical concepts and operations despite normal intellectual functioning and sufficient age-appropriate education (Butterworth, 2004; Shalev, 2004; American Psychiatric Association, 2013). Prevalence of DD has been estimated to be 3–8% of the general population, depending on the identification criteria, and this rate increases up to 13.8% when comorbidities [e.g., attention deficit hyperactivity disorder (ADHD), dyslexia] are present (Barbaresi et al., 2005; Shalev and von Aster, 2008; Butterworth and Laurillard, 2010). Clinical features of developmental dyscalculia include use of “primitive” strategies to solve arithmetic problems with high error rates and long delays, and poor performance in symbolic and non-symbolic magnitude comparison tasks (Geary, 1993; Shalev and Gross-Tsur, 2001; Landerl et al., 2004; Schwenk et al., 2017). Educational achievements in children with DD are typically far behind their peers, and difficulties often continue into adulthood by negatively affecting academic career and quality of life (Gerber, 2012; Wilson et al., 2015). Despite its importance, the neural correlates of DD are still not fully understood.

Numerical abilities have been localized to mainly fronto-parietal, and occipito-temporal regions in the human brain, and alterations in brain functional and/or structural connectivity patterns between these regions have been suggested to be related to DD (Molko et al., 2003; Nieder and Dehaene, 2009; Houde et al., 2010; Arsalidou and Taylor, 2011; Kaufmann et al., 2011; Matejko and Ansari, 2015; Moeller et al., 2015; Sokolowski et al., 2017; Arsalidou et al., 2018; Peters and De Smedt, 2018; Skeide et al., 2020). A widely used *in vivo* approach to study such alterations of structural connectivity is diffusion tensor imaging (DTI) that provides information about white matter (WM) microstructure [i.e., fractional anisotropy (FA), axial, radial and mean diffusivities (AD, RD, and MD); Basser, 1995].

Developmental DTI studies in typically developing (TD) children (samples ranging in age from 6-to-15-year-old) have provided some evidence for the role of association, projection and commissural fibers in numerical abilities. There were positive correlations between FA values in the bilateral superior longitudinal fasciculus (SLF), inferior longitudinal fasciculus (ILF), inferior fronto-occipital fasciculus (IFOF), left arcuate fasciculus (AF), and exact/approximate arithmetic performance (Van Eimeren et al., 2008; Tsang et al., 2009; Li et al., 2013; Van Beek et al., 2014). FA values in the left superior corona radiata (SCR), internal and external capsules bilaterally (IC and EC) were positively correlated with performance on arithmetic and mathematical reasoning tasks (Van Eimeren et al., 2008; Tsang et al., 2009). Interhemispheric connections such as the forceps major, splenium, and isthmus/body of the corpus callosum (CC) were also positively correlated with performance on arithmetic and numerical comparison tasks (Tsang et al., 2009; Cantlon et al., 2011; Li et al., 2013). These studies therefore suggest that higher numerical performance corresponds with increased WM coherence, and lower numerical performance corresponds with decreased WM coherence.

So far, two cross-sectional and one longitudinal DTI studies have compared brain structural connectivity between children with DD and TD peers. 7–9-year-old children with DD showed reduced FA in the right corticospinal tract (CST), right ILF and right IFOF, bilateral anterior thalamic radiation (ATR), bilateral SLF and forceps major and splenium of the CC compared to TD children (Rykhlevskaia et al., 2009). The second cross-sectional study reported reduced FA and increased RD in the left SLF in 10-year-old children with DD compared to TD peers (Kucian et al., 2013). The study following 3–6-year-old children until the age of 7–9 years found that children who developed DD within that period could be distinguished from their TD peers with up to 81% accuracy using the right SLF that showed lower structural connectivity in DD (Kuhl et al., 2021). All of these studies lead us to hypothesize that children with DD would have lower WM coherence than their TD peers in certain tracts.

To our knowledge, no studies have investigated the relationship between tract length and numerical competencies. Compared to previous DTI studies, we now have high angular and spatial resolution diffusion-weighted magnetic resonance imaging (dMRI) that allows us to use advanced crossing-fiber probabilistic tractography methods (Behrens et al., 2007). Tract length can thus be measured more precisely *in vivo* with this method.

This study’s original contribution to the literature is the tract length evaluation of DD and TD groups via probabilistic tractography. Various developmental and aging studies have shown that tracts tend to lengthen from birth to young adulthood relative to the brain size, possibly also due to experience-dependent plasticity, and then shorten again during normal aging (Tang et al., 1997; Marner et al., 2003; Yu et al., 2014; Asaridou et al., 2020). Several studies on healthy aging and/or neuropsychiatric diseases have found that tract length is positively correlated with cognitive functions and negatively correlated with aging and/or disease presence/severity (Buchsbaum et al., 2006; Salminen et al., 2013; Torgerson et al., 2013; Behrman-Lay et al., 2015; Baker et al., 2017; Heaps-Woodruff et al., 2018). Therefore, it seems plausible to hypothesize that dyscalculic children’s impaired numerical abilities correspond with shorter tracts than their TD peers. Shorter fibers in DD might show weaker/altered communication between cortex areas due to altered/delayed fiber maturation/myelination (Fields, 2010; Yu et al., 2014; Almeida and Lyons, 2017; Oishi, 2017).

In this study, we evaluate WM microstructure properties using voxel-wise methods with DTI to replicate previous findings, and the length of the reconstructed tracts using crossing-fiber probabilistic tractography to expand on them. Specifically, we expect that children with DD have lower WM coherence and shorter tracts than their TD peers for certain association (i.e., SLF, ILF, and IFOF bilaterally), projection (i.e., ATR, SCR, IC, and EC bilaterally and right CST) and commissural (i.e., body and splenium of CC and forceps major) fibers.

## 2. Materials and methods

### 2.1. Participants

Non-clinical participants were recruited in a population-based, longitudinal design in three stages from 13 randomly chosen primary state schools representative of low, medium, and high socioeconomic backgrounds in Ankara, the capital of Türkiye (see also Üstün et al., 2021). In the first stage, we screened 2,058 third-grade students from these schools with fluid intelligence (Raven’s Standard Progressive Matrices Test (RSPM; Raven, 2000); Turkish version by Şahin and Düzen (1993)) and mathematics ability tests (curriculum based Mathematics Achievement Test (MAT; Fidan, 2013) and Calculation Performance Test (CPT; De Vos, 1992); Turkish version by Olkun et al. (2013)). The children’s teachers completed attention deficit hyperactivity disorder (ADHD) and learning difficulty questionnaires (Swanson, Nolan, and Pelham, SNAP-IV Questionnaire; reliability and validity by Bussing et al. (2008) and Strengths and Difficulties Questionnaire (SDQ) by Goodman (2001); Turkish version by Güvenir et al. (2008)). In the second stage, children at risk of developmental dyscalculia (DD candidates) and children with normal achievement as typically developing (TD candidates) were determined via inclusion and exclusion criteria according to the results of the screening tests and questionnaires. First, all children with incomplete data or those outside the mean age range of ±2.5SD were excluded, leaving 1,880 possible participants (mean age = 8.66 ± 0.41, 953F, 927 M). We divided the age range (7.5–9.5 years) into four categories of 6-month periods. From this participant pool, we determined DD candidates and subsequently TD candidates by applying inclusion and exclusion criteria within each 6-month age category: DD candidates were those in the lowest 25th percentile of the MAT and CPT scores; TD candidates were chosen randomly between 35 and 75th percentiles of the MAT and CPT scores. Exclusion criteria for all participants included: (1) the lowest 10th percentile of the RSPM scores, and (2) the highest 15th percentile of the SNAP-IV scores.

In the last stage, 2 years later, we re-assessed DD and TD candidates in detail and subsequently we scanned the identified children using MRI. We excluded children who were left-handed, born prematurely (birth weight < 2,500 g and gestation period <36w) or those with psychiatric or neurological diseases. Child and adolescent psychiatrists evaluated volunteer participants to determine psychiatric comorbidities, such as ADHD and anxiety, during semi-structured interviews (Schedule for Affective Disorders and Schizophrenia for School Age Children-Present and Lifetime Version-Turkish Version; Gökler et al., 2004). Expert psychologist tested the children using the Wechsler Intelligence Scale for Children (WISC-R; Wechsler, 1974); Turkish version by Savaşır and Şahin (1995) to exclude those with intellectual disability (total IQ score < 80). We re-tested the children with the MAT and CPT to ensure they were in the previously determined groups. We evaluated reading disability with a time-limited reading test and eliminated dyscalculic children who read less than 80 words per minute (Öner et al., 2019). All of the detailed evaluations led to the exclusion of 12 participants; 14 participants did not agree to the MRI assessment and we were not able to reach all of the candidates in the determined pool. Therefore, we were able to include 12 DD and 16 TD children in the MRI part of the study. We additionally had to exclude two DTI data sets from the DD group as explained in the related section (2.3). Demographic and cognitive profiles of the sample can be seen in Table 1. DD children had significantly lower MAT and CPT scores than TD peers, whereas age, gender, handedness, total WISC-R (corrected for arithmetic), and reading scores were well-matched between the groups.

**Table 1:** Demographics and behavioral/cognitive profiles of the developmental dyscalculia (DD) and typically developing (TD) groups.

	DD	TD
*N*	10	16
Gender (f/m)	8/2^a,e^	9/7^a,e^
Handedness (r/l)	10/0	16/0
Handedness	13.8 (1.1)^b,e^	14.5 (1.8)^b,e^
Age (years)	11.3 (0.7)^b,e^	11.2 (0.6)^b,e^
Age range (years)	10.1–12.2	10.4–12.2
Numerical tests		
MAT	9.1 (2.9)^**b^	20.3 (2.5)^**b^
CPT sum	67 (16.3)^**b^	104.6 (10.7)^**b^
CPT addition	19 (3.8)^**c^	25.4 (3.3)^**c^
CPT subtraction	14.9 (4.7)^*b^	20.4 (2.8)^*b^
CPT multiplication	13.2 (5.1)^**c^	21.6 (2.7)^**c^
CPT division	7.4 (3.8)^**b^	16.6 (2.8)^**b^
CPT mix	12.5 (3)^**b^	20.5 (3.1)^**b^
IQ tests		
WISC-R performance	98.6 (9.7)^b,e^	103.3 (11)^b,e^
WISC-R verbal	88.3 (9)^**b^	106.6 (10.5)^**b^
Arithmetic sub test (from verbal IQ)	6.5 (2.1)^*b^	10.6 (2.9)^*b^
WISC-R total	92.5 (8.1)^*b^	105.7 (9.6)^*b^
WISC-R total (corrected for arithmetic)	95.6 (SE = 3.2)^d,e^	103.8 (SE = 2.4)^d,e^
Reading evaluation	101.6 (12.9)^c,e^	106.7 (25.7)^c,e^

MAT, Mathematics achievement test; CPT, Calculation performance test; WISC-R, Wechsler intelligence scale for children. Scores are given as mean where appropriate. ^a^Fischer-Chi-Square-test, ^b^Independent-samples *t*-test, ^c^Mann-Whitney-U-test, ^d^ANCOVA. ^*^p < 0.05, ^**^p < 0.001, ^e^*p* > 0.05. Detailed information about the tests used in the study can be found here (Öner et al., 2018; Üstün et al., 2021).

Behavioral tests (MAT, CPT, RSPM, SNAP IV, and SDQ Questionnaire) used in this study were described in detail by Öner et al. (2018).

### 2.2. MRI data acquisition

Participants were familiarized with the MRI procedure in a mock scanner. We acquired anatomical and dMRI data on a 3 Tesla Tim-Trio MRI scanner (Siemens, Erlangen, Germany) using a 16 channel head-coil. Children watched cartoons via the projective mirror system during both acquisitions. High-resolution diffusion-weighted images were obtained along 60 different directions with a *b*-value of *b* = 700 s/mm^2^ and 10 initial reference images with *b* = 0 s/mm^2^. The images were acquired with anterior-to-posterior phase encoding direction containing 75 slices in transversal orientation without gap. An echo-planar imaging sequence was used via interleaved recording. Other scan parameters were: TR: 9.506 s, TE: 85 ms, FOV: 198 mm, Flip Angle: 90, voxel size 1.55 m × 1.55 mm × 1.55 mm, single spin echo. The dMRI acquisition lasted 11 m 35 s. High-resolution structural T1-weighted image acquisition parameters were: TR: 2.6 s, TE: 3.02 ms, FOV: 256 mm, matrix: 256 × 256 providing an isotropic resolution of 1 mm.

### 2.3. Diffusion-weighted MRI data preprocessing

Preprocessing was implemented in a Nipype-workflow (Gorgolewski et al., 2011). First, dMRI images were preprocessed with the MRtrix3 (Tournier et al., 2019) DWI-denoising tool to suppress local signal fluctuations due to thermal noise (Veraart et al., 2016a,b). Then we corrected the data for eddy-current induced distortions and subject head motions with the “eddy” tool (Andersson and Sotiropoulos, 2016) implemented in FSL (v6.0.1; Smith et al., 2004). Simultaneously, we performed slice-to-volume correction (Andersson et al., 2017), and outlier detection and replacement (Andersson et al., 2016). Because children, especially those with developmental difficulties tend to move more during MRI (Yendiki et al., 2014; Dosenbach et al., 2017), we closely controlled those modern and highly advanced preprocessing options of optimal alignment performing FSL-based automated quality control analysis after preprocessing (Bastiani et al., 2019). According to the quality control results, we excluded two data sets with the highest motion parameters and outlier replacement percentage, as well as the lowest SNR/CNR, resulting in more balanced subject motion for groups [see the independent samples *t*-test results and detailed information in Supplementary Table S1, Supplementary Figure S1, Supplementary Notes S1, S2]. Individual brain masks were created using FSL Brain Extraction Tool (Smith, 2002).

We used FSL Diffusion Tool to obtain FA, AD, RD, and MD maps. FA represents the degree of anisotropic diffusion in tissue (Alexander et al., 2007). While MD measures the average diffusivity in tissue, AD represents the diffusivity along main fiber direction, and RD describes the average diffusivity perpendicular to the axonal axis. These DTI metrics are commonly used to characterize WM microstructure. They are influenced by different microstructural properties such as axonal density, WM coherence and/or maturation, intra-voxel orientation dispersion, myelination, membrane permeability, or number of axons (Feldman et al., 2010; Jones et al., 2013). Therefore, tracts with a denser axonal packing and higher myelinations tend to have higher FA and lower diffusivity values, and are often interpreted as higher WM coherence relating to faster and more efficient transmission of the axonal signals compared to less densely packed tracts.

### 2.4. White matter microstructure analysis

We inspected WM microstructure with individual FA, MD, RD, and AD maps performing voxel-wise analysis with Tract-Based Spatial Statistics (TBSS; Smith et al., 2006). We performed TBSS with its standard steps focusing on certain regions-of-interest (ROIs). First, FA images were aligned to each other and to the MNI152 standard space by applying combined non-linear and linear registration algorithms. Following that, all images were averaged, and the mean FA skeleton mask was created using a threshold of 0.2. Finally, individual skeletonized FA, MD, RD, and AD maps were obtained for further analyses.

Based on the previous literature, we selected SLF, ILF, IFOF, ATR, SCR, IC, and EC bilaterally, right CST, body and splenium of the CC and forceps major as ROIs. We defined the ROIs using the two Johns-Hopkins-University atlases in FSL with a threshold of 25%, yielding the core of each of the tracts. We masked each thresholded ROI and multiplied it by the mean FA skeleton mask. The acquired ROI masks were then used to compare the individual skeletonized FA and diffusivity maps between two groups.

The FSL tool “randomise” was used for the permutation tests of the TBSS (Winkler et al., 2014). We conducted independent samples *t*-test for each ROI with 10,000 permutations to assess the group differences. Threshold-Free Cluster Enhancement (TFCE) was employed as the clustering method (Smith and Nichols, 2009). Statistical significance value was defined as *p* < 0.05 and corrected for multiple comparisons with Family-Wise Error correction (FWE). The contrast of Controls > Dyscalculics demonstrated which WM tracts showed higher FA, MD, RD, and AD values in TD controls than children with DD. The contrast of Dyscalculics > Controls demonstrated vice versa.

### 2.5. Tract length analysis

We used probabilistic tractography to assess the length of the pathways, because the method has the advantage of taking noise and fiber spreading into account as uncertainty and better in dealing with crossing fibers in a voxel by calculating the most likely connections from estimated fiber orientation distribution (Behrens et al., 2007; Tournier et al., 2011). We computed the fiber orientation distribution using the FSL tool BEDPOSTX with up to two fiber directions per voxel using the ball-and-two-stick model (Behrens et al., 2007). The WM pathways were then automatically reconstructed using the TRACULA software (Yendiki et al., 2011), which is based on Bayesian framework for global probabilistic tractography (Jbabdi et al., 2007). Because TRACULA uses the FreeSurfer (v6.0; Fischl, 2012) parcellation and segmentation as prior, we checked the quality of the FreeSurfer outputs and corrected the errors with suitable adjustments following the software recommendations. For the tractography analysis in TRACULA, we used the Freesurfer “bbregister” affine registration algorithm for individual preprocessed diffusion-weighted images to the parcellated-segmented T1-weighted images as intra-subject registration, and FSL “FLIRT” linear (affine) registration algorithm the warped T1-weighted images to the MNI152 template as inter-subject registration.

We focused on the tracts related to numerical abilities as specified above sections, which were also possible to reconstruct within TRACULA software. Therefore, individual ATR, SLF (temporal and parietal parts), and ILF bilaterally, right CST and forceps major fibers were reconstructed. The length of the highest probability path of these selected tracts were measured. The length of the pathway along the center voxel with the highest probability represents the most representative part of the connection and integrates the complex microstructure of the pathway by probabilistic tractography beyond the voxel level. We then compared the length of the pathways between the groups with ANCOVA in SPSS (v24) using the estimated total intracranial volumes (eTIV), which were calculated in FreeSurfer from the T1-weighted images, as a covariate to correct for head sizes. Significant value of *p* was defined as 0.05 for the ANCOVA analyses.

### 2.6. Correlation plots

To illustrate the relationships between WM connectivity measures and mathematical test scores (MAT), we plotted correlation lines using SPSS (v24) in the whole sample. As WM connectivity measures, we used FA, AD, MD, and RD values from the TBSS-ROI analyses and tract length values from the TRACULA probabilistic tractography analyses, both of which showed significant group differences.

## 3. Results

### 3.1. White matter microstructural differences between groups

**Figure 1:**
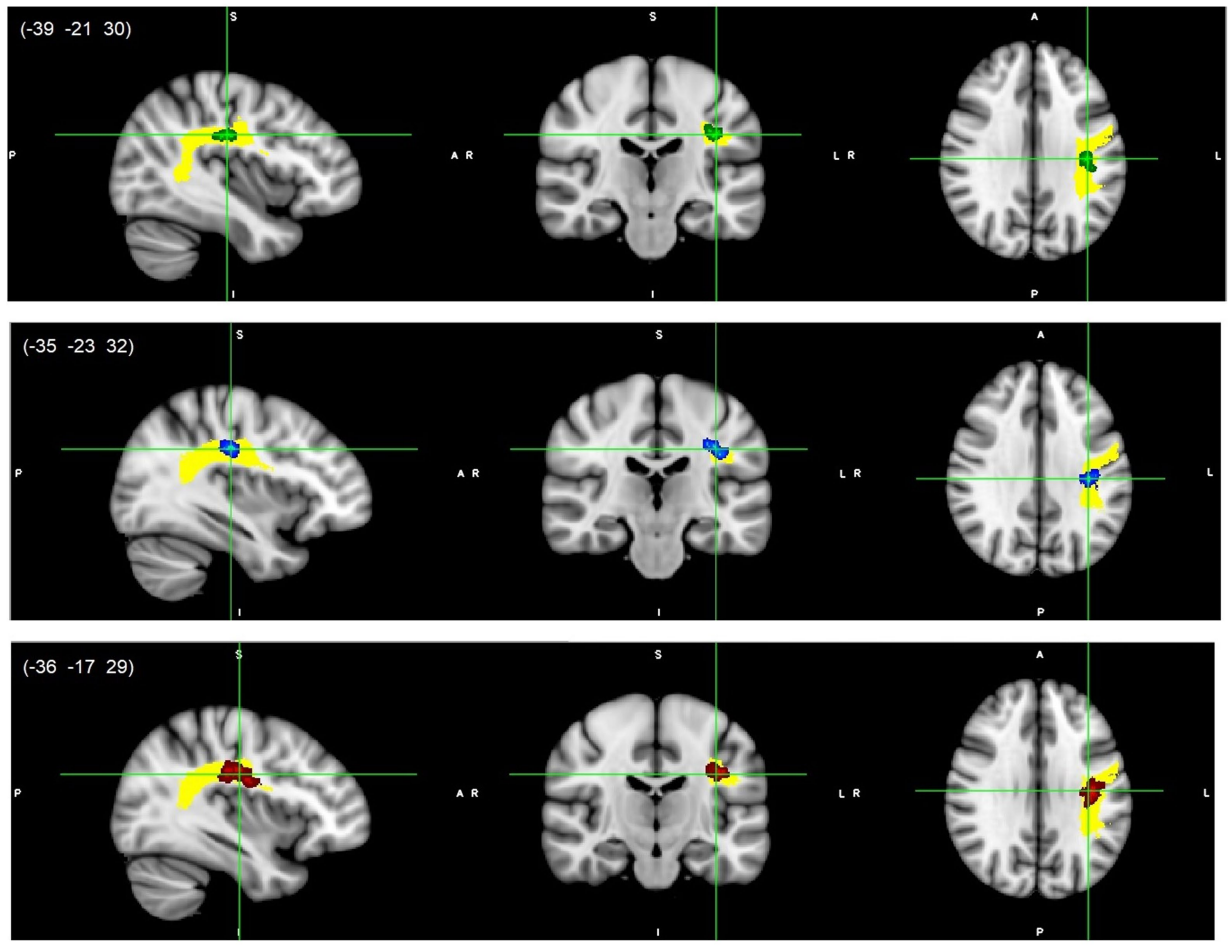
Left SLF/AF white matter microstructure differences. Lower FA (green, top), higher RD (blue, middle), and higher MD (red, bottom) values in DD compared to TD children, *p* < 0.05, FWE corrected with TFCE. Results are thickened with tbss_fill. Yellow region represents selected SLF/AF mask as ROI. SLF/AF, superior longitudinal fasciculus/arcuate fasciculus.

**Figure 2:**
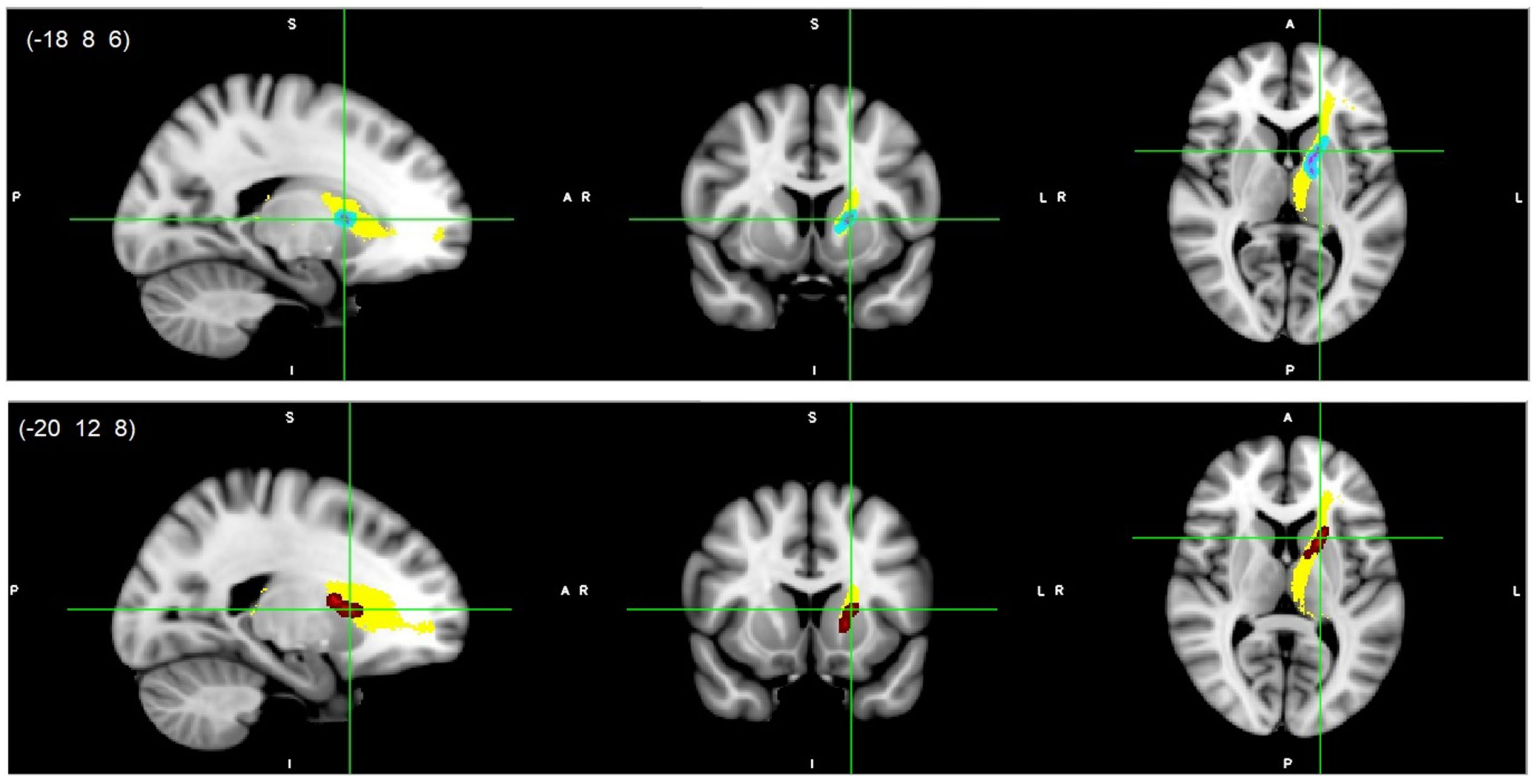
Left ATR white matter microstructure differences. Higher AD (light blue-pink, top) and higher MD (red, bottom) values in DD compared to TD children, *p* < 0.05, FWE corrected with TFCE. Results are thickened with tbss_fill. Yellow region represents selected ATR mask as ROI. ATR, Anterior thalamic radiation.

**Figure 3:**
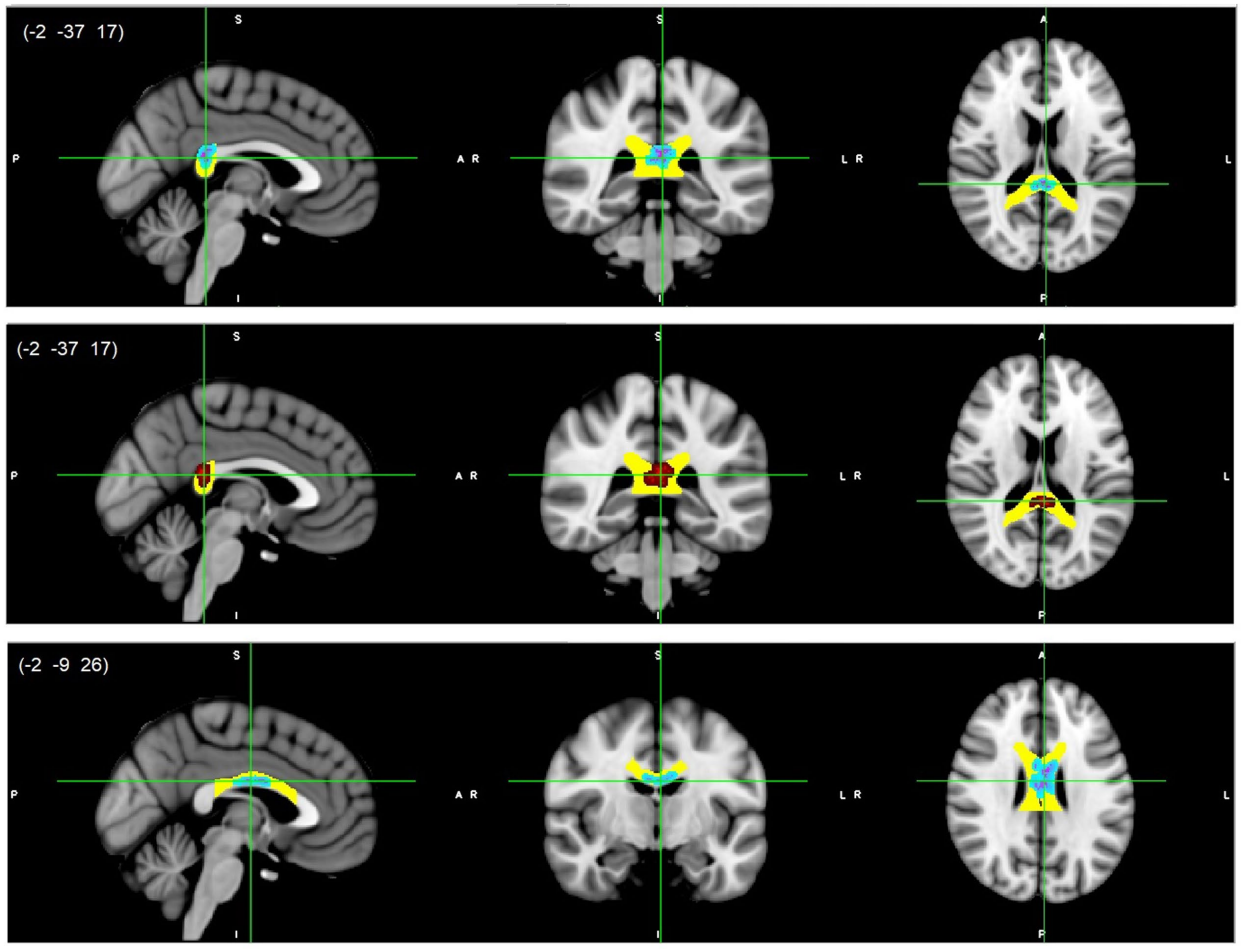
CC splenium (top/middle) and body (bottom) white matter microstructure differences. Higher AD (light blue-pink, top and bottom) and higher MD (red, middle) values in DD compared to TD children, *p* < 0.05, FWE corrected with TFCE. Results are thickened with tbss_fill. Yellow regions represent selected CC masks as ROIs. CC, Corpus callosum.

All reported results here have been found as statistically significant at the *p* < 0.05 level with FWE-correction with TFCE as described above (Table 2; Figures 1–3; Supplementary Figures S2, S3). First, we found lower FA and higher RD, MD, and AD values in DD compared to TD group for the left SLF/AF (Table 2; Figure 1). AD and MD values were higher in DD compared to TD group for the left ATR, CC-splenium, and bilateral IC pathways (Table 2; Figures 2, 3; Supplementary Figures S2, S3). The body of the CC also showed higher AD values in DD than TD group (Table 2; Figure 3, bottom). FA values within the right SCR were lower in DD than in TD (Table 2; Supplementary Figure S2, bottom). Finally, small significant differences were also apparent between groups for the left SCR (RD values) and right ATR (AD values). There were no significant WM microstructural group differences in the bilateral IFOF, bilateral ILF, right SLF, right CST, bilateral EC, and forceps major ROIs.

**Table 2:** TBSS-ROI results: white matter microstructure differences between children with DD and TD controls.

Pathway	Laterality	DTI-measure	Contrast	Voxels	Peak value of p^*^	Peak MNI Coordinates (mm)			Figures
						X	Y	Z	
SLF/AF	L	FA	Controls > Dyscalculia	87	0.023	−38	−23	29	Figure 1
SLF/AF	L	MD	Dyscalculia > Controls	256	0.016	−32	−19	31	
SLF/AF	L	RD	Dyscalculia > Controls	72	0.035	−34	−25	35	
SLF/AF	L	AD	Dyscalculia > Controls	5	0.046	−37	−14	31	-
ATR	L	AD	Dyscalculia > Controls	195	0.003	−18	8	7	Figure 2
ATR	L	MD	Dyscalculia > Controls	151	0.02	−11	−3	2	
CC-Splenium	Bilateral	AD	Dyscalculia > Controls	161	0.008	−1	−36	17	Figure 3
CC-Splenium	Bilateral	MD	Dyscalculia > Controls	121	0.021	−7	−38	20	
CC-Body	Bilateral	AD	Dyscalculia > Controls	198	0.025	−1	−12	26	
IC	R	AD	Dyscalculia > Controls	352	0.016	15	−7	0	Figure S2
IC	R	MD	Dyscalculia > Controls	294	0.016	22	−7	12	
IC	L	AD	Dyscalculia > Controls	197	0.02	−10	−2	1	Figure S3
IC	L	MD	Dyscalculia > Controls	77	0.036	−19	3	12	
SCR	R	FA	Controls > Dyscalculia	88	0.024	27	−11	20	Figure S2
SCR	L	RD	Dyscalculia > Controls	14	0.048	−25	−9	33	-
ATR	R	AD	Dyscalculia > Controls	4	0.047	17	7	7	-

* Corrected for multiple comparisons with FWE correction at *p* < 0.05 level with TFCE method.

FA, Fractional anisotropy; AD, Axial diffusivity; MD, Mean diffusivity; RD, Radial diffusivity; SLF/AF, Superior longitudinal fasciculus/arcuate fasciculus; ATR, Anterior thalamic radiation; CC, Corpus callosum; IC, Internal capsule; SCR, Superior corona radiata.

### 3.2. Tract length differences between groups

**Figure 4:**
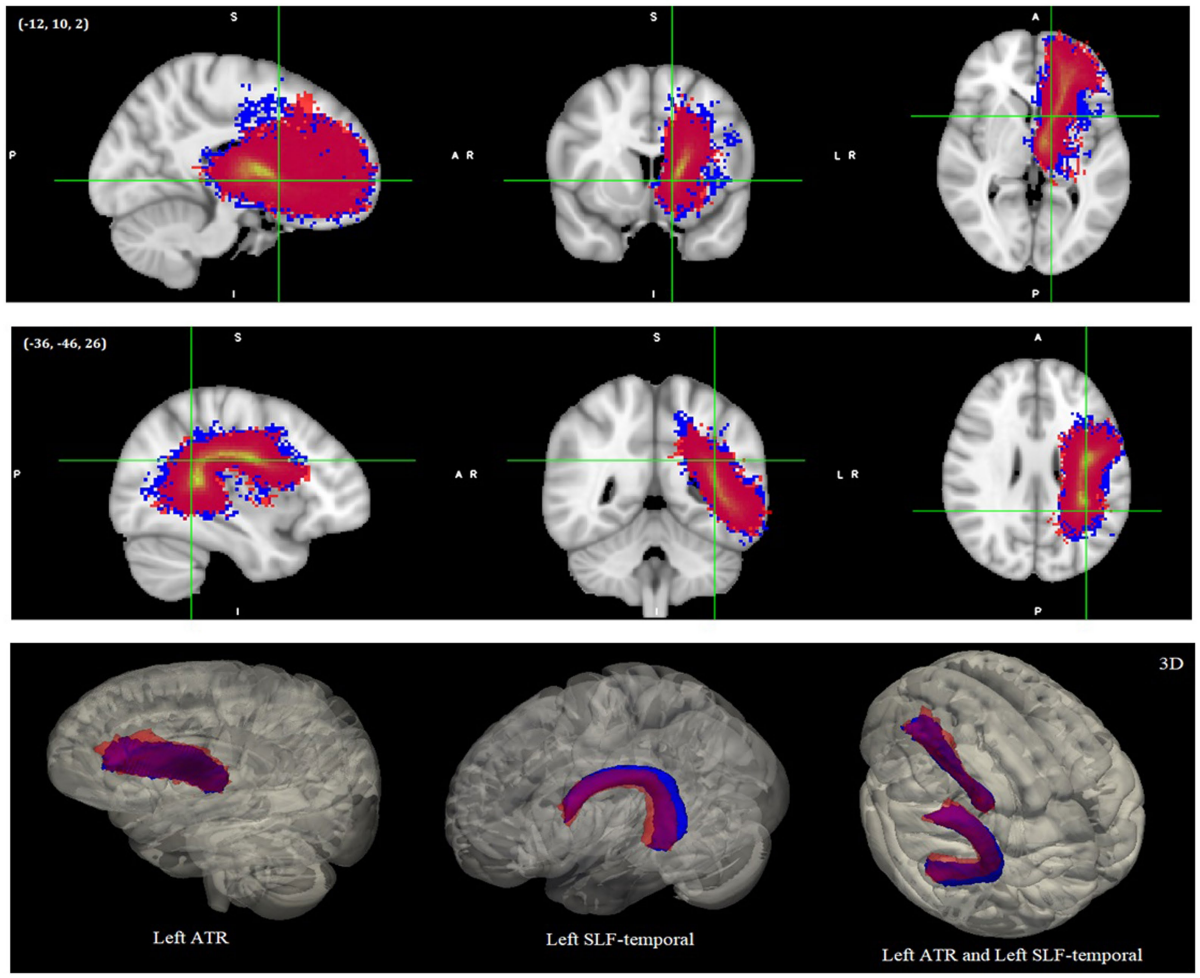
Reconstructed pathways of the DD and TD groups after global tractography. The left ATR-top and left SLF-temporal/AF-middle as 2D images. The 3D versions are shown at the bottom. All images were normalized with their maximum intensity values, and the 2D images were thresholded between 0 and 1 and the 3D images thresholded between 0.2 and 1. Red colors represent DD and blue colors represent TD samples. Images shown here are on MNI152 template. ATR, Anterior thalamic radiation; SLF/AF, Superior longitudinal fasciculus/arcuate fasciculus.

Only two of the reconstructed tracts showed significant tract length differences between the groups. The left ATR and left SLF-temporal/AF were significantly shorter in the DD than TD controls when eTIV was controlled [*F*(1,23) = 6.26, *p* = 0.02, partial η^2^ = 0.21 (Estimated Mean ± SE was 80.13 mm ± 0.99 for TD and 76.08 mm ± 1.26 for DD) for the left ATR and *F*(1,23) = 6.35, *p* = 0.019, partial η^2^ = 0.22 (Estimated Mean ± SE was 92.94 mm ± 1.42 for TD and 87.06 mm ± 1.81 for DD) for the left SLF-temporal/AF]. The Estimated Mean Difference ± SE was −5.88 mm ± 2.33 for the left SLF-temporal/AF (i.e., corrected for eTIV) and the Estimated Mean Difference ± SE was −4.06 mm ± 1.62 (i.e., corrected for eTIV) for the left ATR. Actual means and the standard deviations can be seen in Supplementary Note S3. Reconstructed pathways of the left SLF-temporal/AF and left ATR for each group and tract length differences between groups can be seen in Figure 4 with both 2D and 3D versions. We depicted the endpoints (i.e., two termination or seed regions for tract reconstruction in TRACULA) of the reconstructed pathways for each group (see Supplementary Figures S4, S5). We additionally calculated the center of gravity coordinates of the two endpoints for each group to have a better picture of the tract’s shape/trajectory. Then we looked at the distances between the endpoints’ coordinates to examine how the TD group’s endpoint coordinates for a certain tract were positioned relative to the DD group’s endpoint coordinates for that tract (see Supplementary Table S2 and Supplementary Note S5). No significant group differences were found in the right SLF-temporal/AF, bilateral SLF-parietal, right ATR, bilateral ILF, right CST, and forceps major tracts. See Supplementary Note S3 for the results of the ANCOVA tests, means and standard deviations of the tract length values for other tracts.

### 3.3. Correlation plots between structural connectivity and behavioral measures

**Figure 5:**
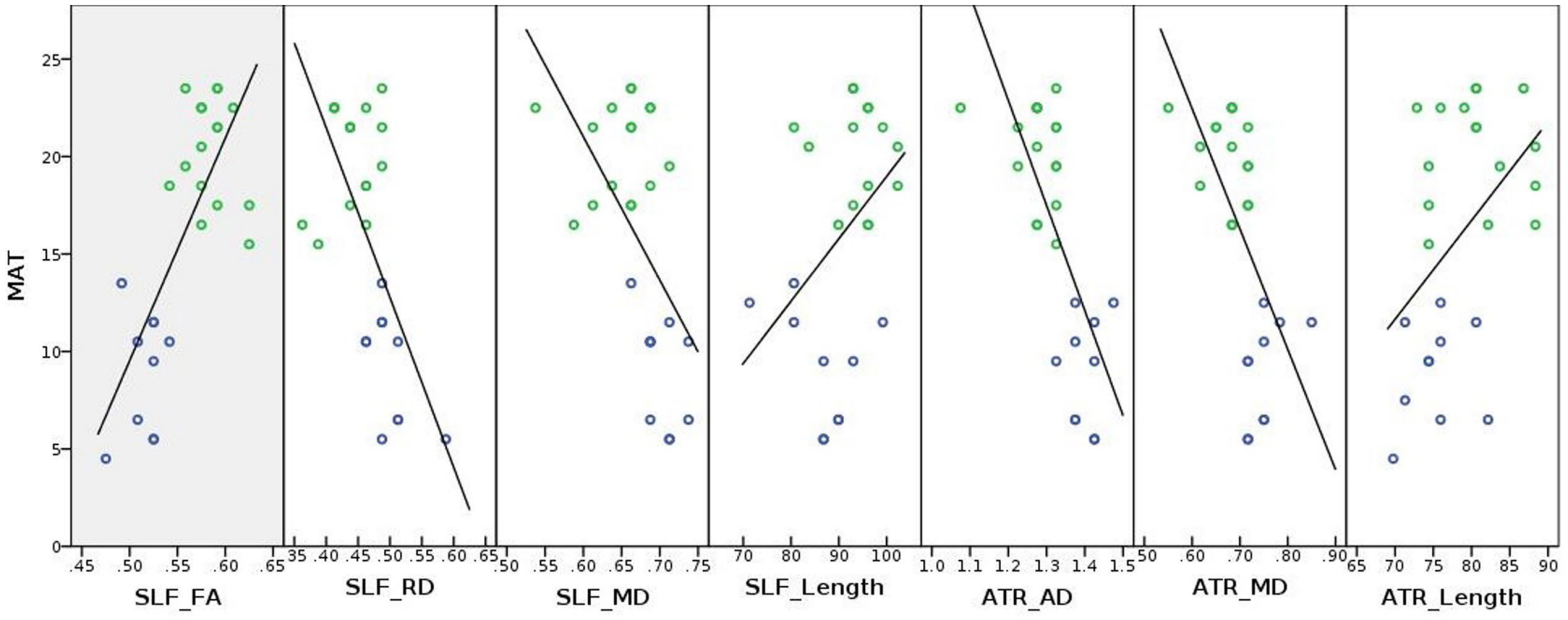
Correlation lines were plotted between white matter coherence (i.e., DTI derived FA, AD, MD, and RD values), tract length, and math achievement for illustrative purposes. MAT, Math achievement test; ATR, Anterior thalamic radiation; SLF, Superior longitudinal fasciculus; FA, Fractional anisotropy; AD, Axial diffusivity; MD, Mean diffusivity; RD, Radial diffusivity; blue and green circles represent developmental dyscalculia and typically developing groups, respectively; and Unit of the diffusivity values: 10^−3^ mm^2^/s, Unit of the tract length: “mm.”

The plotted correlation lines between math scores, tract length values (in “mm”) and DTI derived indices are shown in Figure 5. As shown in Figure 5, FA values of the left SLF-temporal/AF seemed to be positively correlated with the mathematics test scores, whereas diffusivity values (AD, MD, and/or RD) of both the left SLF-temporal/AF and left ATR tracts seemed to be negatively correlated with the mathematics test scores. The tract lengths of the left SLF-temporal/AF and left ATR tended to be positively correlated with the mathematics test scores.

## 4. Discussion

This study aimed to identify structural connectivity alterations in DD compared to TD peers. The WM microstructure analysis showed that children with DD had lower structural coherence, and the tract length analysis provided the first evidence that children with DD had shorter tracts. We also illustrated that WM coherence and tract length measures tended to be positively correlated with math performance, which may be important to explore the link between imaging biomarkers and behavior in numerical development. In the following, we discuss the results of our regional analyses targeting major fiber tracts related to numerical disabilities.

### 4.1. Association fibers

We found lower FA, higher RD, and higher MD values exclusively in the left SLF/AF in children with DD compared to TD peers. Our study partially confirmed the findings of the previous studies, which showed lower FA and/or higher diffusivities in the bilateral SLF in 7–9 or 10-year-old children with DD compared to TD peers (Rykhlevskaia et al., 2009; Kucian et al., 2013; Kuhl et al., 2021). In line with this, math-gifted adolescents had higher FA in the bilateral SLF, and impaired arithmetic competencies in other clinical conditions in children and adolescents were linked to lower FA in the left SLF (Barnea-Goraly et al., 2005; Lebel et al., 2010; Till et al., 2011; Navas-Sanchez et al., 2014). Our findings were also consistent with previous studies in TD children of various ages (ranging from 7-to-15-years-old) and in young adults, which showed positive correlations between WM coherence in the left SLF/AF and numerical abilities (Tsang et al., 2009; Klein et al., 2013; Li et al., 2013; Matejko et al., 2013; Van Beek et al., 2014; Jolles et al., 2016).

Moreover, we found a shorter left SLF-temporal/AF in the DD than in the TD group. When we examine the reconstructed left SLF-temporal/AF (see Figure 4 and Supplementary Figure S4), the tracts between the groups seem to have different trajectories, which can explain the tract length differences between the groups. For a better explanation of how the tracts’ shapes/trajectories vary between the groups, we computed the center of gravity coordinates between the two endpoints of the groups as well as the distances between them (see Supplementary Table S2 and Supplementary Note S5). The TD group’s frontal, particularly, the temporal endpoints seem to be more dorsal-posterior than the DD group, resulting in a larger bending angle within the SLF-temporal/AF and hence longer tracts. Whereas the DD group’s endpoints seem to be more ventral-anterior than the TD group, resulting in a smaller bending angle within the SLF-temporal/AF and hence shorter tracts. The length of the minimum and maximum tracts within the DD group’s SLF-temporal/AF bundle tend to be also shorter than the TD (see Supplementary Note S4 for the minimum and maximum tract length values). Shorter tracts in DD might be related to a developmental delay in the white matter, i.e., altered/delayed axonal maturation/myelination and the tracts connecting frontal and SMG are also known to take more time to develop in childhood (Zhang et al., 2007; Fields, 2010; Almeida and Lyons, 2017). There might also be a developmental delay in the gray matter (Rakic, 2009; Zatorre et al., 2012). Supporting this, recently we showed less volume and surface area in the left fronto-parietal cortex (i.e., precentral gyrus and SMG) in the DD group (Ayyıldız et al., 2023). Weaker WM coherence seems to match with shorter tracts in the DD group. One interpretation would be that the growth/maturing/experience related plastic changes of the fibers in line with the gray matter development would result in an increase in tract length due to increasing size and numbers of neurons and supportive cells (Rakic, 2009; Fields, 2010; Zatorre et al., 2012; Almeida and Lyons, 2017; Asaridou et al., 2020). If there is a developmental delay in white matter, the coherence strength and the fiber length can be expected to match and delay together. On the other hand, shorter tracts can also be a result of a weaker WM coherence. Longitudinal and interventional developmental studies are required to test these intriguing hypotheses.

The SLF connects frontal regions to parietal, temporal, and occipital regions (Makris et al., 2005; Schmahmann et al., 2008; Conner et al., 2018). The fronto-parieto-temporal part of the SLF is also known as AF and is additionally involved in the dorsal stream of the language network (Catani et al., 2005; Anwander et al., 2007; Brauer et al., 2011; Wang et al., 2016; Eichert et al., 2019). The dorsal language network is related to the phonological aspects and verbal working memory processes of language (Hickok and Poeppel, 2004; Friederici and Gierhan, 2013). In addition to the bilateral intraparietal sulci and bilateral superior parietal lobules for number processing, concordant with the dorsal language network, verbal numerical processing has been attributed to the left perisylvian language regions (Dehaene et al., 2003; Dehaene, 2011; Smaczny et al., 2023). Task-based fMRI meta-analyses have shown the fronto-parieto-temporal network is involved in the brain’s numerical functions (Houde et al., 2010; Arsalidou and Taylor, 2011; Kaufmann et al., 2011; Sokolowski et al., 2017; Arsalidou et al., 2018). Present results showing both reduced WM coherence and shorter length of the left SLF/AF therefore might be associated with verbal number processing problems in DD.

### 4.2. Projection fibers

We found lower FA in the right SCR, higher RD in the left SCR (in a relatively small cluster) and higher AD and MD in the bilateral IC in DD compared to TD peers. In line with our findings, FA values in the left or bilateral SCR and IC showed positive correlations with arithmetic performance in TD children (Van Eimeren et al., 2008; Tsang et al., 2009). Similarly, one study showed that math training reduced RD in TD children, and the other one showed lower FA in children with comorbid-dyscalculia (Lebel et al., 2010; Hu et al., 2011). IC and SCR extend throughout the sensory-motor pathways from/to high-level cognitive brain areas (FitzGerald et al., 2012). Several studies show that sensory-motor pathways could be related to finger counting (Noel, 2005; Sato et al., 2007). Therefore, differences in IC and SCR might be related to deficiencies in DD regarding the use of counting strategies in arithmetical problem-solving. We found that AD and MD in the left ATR were larger in the DD than in the TD group. Similar to our findings, children with DD showed lower FA in the bilateral ATR, and math-gifted adolescents showed higher FA compared to their TD peers (Rykhlevskaia et al., 2009; Navas-Sanchez et al., 2014).

Furthermore, we found a shorter left ATR in the DD group. When we examine the reconstructed left ATR between the groups (see Figure 4 and Supplementary Figure S5), the TD group appears to have more consistent tracts, whereas the DD group appears to have more irregular tracts that fan out to the prefrontal cortex (e.g., to the anterior, dorsolateral, and superior parts) and appear earlier in their course. We also compared the distances of the center of gravity coordinates of the two endpoints for a better understanding of the tracts’ variations of the groups. Although the positions of the anterior thalamic endpoints of the ATR appear to be similar between the groups, the prefrontal endpoints appear to be more ventral-inferior in the TD group than in the DD (see Supplementary Figure S5, Supplementary Table S2, and Supplementary Note S5). The length of the maximum and especially the minimum tracts within the DD group’s ATR bundle tend to be also shorter than the TD group (see Supplementary Note S4 for the minimum and maximum tract length values). Therefore, the DD group seems to have less long and/or less organized tracts reaching the prefrontal cortex that might be related to altered/delayed fiber maturation/myelination, as well as the different developmental stages of the prefrontal cortex from birth through young adulthood (Rakic, 2009; Kolb et al., 2012; Yu et al., 2014; Chini and Hanganu-Opatz, 2021). Our task-based fMRI and surface-based morphometry findings with the same cohort, which showed left frontal-hippocampal hyper-activation and lower frontal gray matter volume and surface area in the DD group compared to the TD, indicating that similar brain regions appear to be impaired in DD (Üstün et al., 2021; Ayyıldız et al., 2023).

The ATR has reciprocal connections from/to the anterior nuclei of the thalamus, which is a conjunction hub for the connections between hippocampus and mammillary bodies, to/from the (pre) frontal as well as anterior cingulate cortices (Behrens et al., 2003; Child and Benarroch, 2013). This tract has been shown to be related to learning, episodic, and spatial memory and executive functions of the brain (Vanderwerf et al., 2003; Aggleton et al., 2010; Jankowski et al., 2013; Dillingham et al., 2015). Our findings showing both reduced WM coherence and shorter length in the left ATR might be linked to number-related learning, memorizing and processing issues in DD.

### 4.3. Commissural fibers

We found higher AD and RD in the body and splenium of the CC in the DD compared to the TD group. Consistent with our findings, children with DD or individuals with poor math skills in other clinical conditions showed lower FA in the splenium/body of the CC (Rykhlevskaia et al., 2009; Lebel et al., 2010; Till et al., 2011). Some studies also found positive correlations between FA in the splenium/body of the CC and math/arithmetic performance in TD children, higher FA in math-gifted adolescents and in math-trained samples compared to their TD peers (Tsang et al., 2009; Cantlon et al., 2011; Hu et al., 2011; Navas-Sanchez et al., 2014). The splenium of the CC primarily connects temporo-parietal and also occipital lobes; the body of the CC connects primary and secondary sensory-motor cortices (Aboitiz et al., 1992; Gazzaniga, 2000; Knyazeva, 2013). Differences in bilateral sensory-motor connectivity and the reduced fronto-parieto-temporal connectivity in our study might contribute to a reduced interhemispheric communication in DD in the body and splenium parts, respectively.

### 4.4. Relationships between brain structure and function in developmental dyscalculia

As mentioned above, previously, we showed that fronto-parietal, hippocampal-prefrontal, and occipito-temporal BOLD activations during symbolic and non-symbolic numerical comparison tasks both in the DD and TD groups (Üstün et al., 2021) as well as in adults (Vatansever et al., 2020). Some studies with children as well as adults with DD showed greater activation and altered functional connectivity patterns during numerical comparison and arithmetic problem solving tasks within the regions related to number processing (Rosenberg-Lee et al., 2015; Bulthe et al., 2019; Üstün et al., 2021). Additionally, some structural brain studies using T1-weighted anatomical imaging, including our recent study with the same cohort, tended to show lower gray and/or white matter volumetric changes in the numerical networks (Rotzer et al., 2008; Ranpura et al., 2013; Kuhl et al., 2020; McCaskey et al., 2020; Michels et al., 2022; Ayyıldız et al., 2023). Therefore, our DWI findings having especially weaker and/or atypical SLF/AF and ATR fibers together with structural gray matter differences in the numerical networks might underlie altered functional (connectivity) patterns in children with DD when compared to TD peers.

### 4.5. Limitations, and suggestions for future research

We recruited our sample in a consistent way using a population-based longitudinal design; however, this resulted in a small sample size that is the main limitation of our study. Unfortunately, we were unable to reach all selected children because of some external reasons.

There has been no established gold standard to diagnose DD in children in the field (von Aster and Shalev, 2007; Shalev and von Aster, 2008; Fias et al., 2013; Kucian and von Aster, 2015; Castaldi et al., 2020). Some variations in our findings showing from previous DWI studies, thus, can be caused by different diagnostic criteria for DD used between the studies.

We excluded children with low reading and IQ scores evaluated by the experts from the DD sample. However, the DD group still showed lower verbal IQ scores than the TD group (see Table 1); this might be a confounder in our results. Another confounder might be socioeconomic status of the children because it can affect the brain development and/or math development in the brain (Noble et al., 2005, 2012; Demir-Lira et al., 2016; Rosen et al., 2018; Rakesh et al., 2023). Although we tried choosing our sample from the representative schools from low-middle-high socioeconomic areas, we did not specifically assess it. Therefore, the differences in the DD group might be also related to atypical brain development or there might be similar/shared problems with other neurodevelopmental difficulties like dyslexia (Peters et al., 2018).

Some studies have suggested that DD could be investigated with subtypes as pure DD (i.e., domain-specific), DD with genetic origin, DD with comorbidities with reading, attention and (visuospatial) working memory problems (i.e., domain-general), slight or severe DD (von Aster, 2000; Andersson, 2010; Geary, 2011; Szucs, 2016). One of the criterion to define our DD group was being in the normal norms of the Raven Standard Progressive Matrices and (performance and verbal) IQ tests, which include verbal and visuospatial tasks; however, we had no separate standard test to evaluate working memory in children. Additionally, there have been studies suggesting different brain circuits for verbal and visual number processing, i.e., magnitude comparison and arithmetic problem solving tasks including exact (fact retrieval) and approximate calculations (Dehaene, 1992; Dehaene and Cohen, 1995; Amalric and Dehaene, 2019). Similarly, possible subtypes of DD can be reflected by some deficits/alterations in common and/or dissociable numerical networks in the brain as previously suggested (Stanescu-Cosson et al., 2000; Holloway et al., 2010; Lyons et al., 2015; Klein and Knops, 2023). We had no focus on any possible DD subtypes in our study. More neuroimaging studies are required to further explore brain networks that may be specific to certain numerical tasks in the developing brain, allowing for the classification and diagnosis of possible subtypes of DD.

For TBSS-ROI analyses, defining threshold as 25% of the maximum intensity values of each tract might cause to some missing voxels in the analysis. We wanted to follow a consistent and non-subjective probabilistic tractography method throughout the study to reconstruct and evaluate the tract length. Using Freesurfer parcellations-segmentations as anatomical priors within global tractography approach seemed more reliable to us for the child brain. However, we could not separately evaluate the length of the bilateral IFOF, IC, EC, SCR, splenium and body of the CC as ROIs from the TBSS-ROI analysis because TRACULA had no specific implementation for these pathways. Instead, TRACULA in its fiber reconstruction dictionary reconstructs ILF overlapping with IFOF; CST partially overlapping with IC, EC and SCR; and forceps major including the splenium and body (isthmus) parts of the CC. However, future research should be aware of this limitation.

Especially in childhood, neuroplasticity in the white matter is as important as in the gray matter as learning like experiences affect myelination in the brain (Zatorre et al., 2012). There are some DWI studies on plasticity changes in the brain during a task examined by diffusivity parameters (Scholz et al., 2009; Sagi et al., 2012; Sampaio-Baptista and Johansen-Berg, 2017) and resting state fMRI studies on white matter functional connectivity examined by low-fluctuated BOLD signal (Li et al., 2020; Wang et al., 2022). Future studies can try both methods for a better investigation of the brain structure–function coupling to study neuro-plastic changes in numerical (dis)abilities. The MRI methods including DWI constantly being improved with advancing technology; however, one should carefully interpret the results, as the behavioral outputs still cannot be attributed to the true/exact biological processes in the brain.

## 5. Conclusion

We studied structural connectivity related to DD in children. One main finding of our study—reduced WM coherence and shorter length of the left superior-longitudinal/arcuate-fasciculus—might be related to deficiencies in verbal aspects of number processing in DD. The other major finding—reduced WM coherence and shorter length of the left anterior thalamic radiation—might be attributed to insufficiencies in encoding, retrieving and working with arithmetical facts in children with DD. Left lateralized differences might be related to hemispheric dominance in arithmetic processing during development (Artemenko et al., 2020). Although we had a small sample size, we replicated the finding that lower WM coherence in association, projection, and commissural fibers is related to DD, which is consistent with previous literature. However, the exploratory tract length finding needs to be replicated in new datasets. Therefore, further studies are necessary to investigate DD in children with larger datasets, using longitudinal and interventional designs, to confirm our findings in the future.

## Declaration

Preprint version of this manuscript can be found here: Ayyıldız et al. (2021).

## Data availability statement

The raw data supporting the conclusions of this article will be made available by the authors, without undue reservation.

## Ethics statement

The studies involving humans were approved by Ethical Committee of the Faculty of Medicine at Ankara University (Decision No: 03–95-14). The studies were conducted in accordance with the local legislation and institutional requirements. Written informed consent for participation in this study was provided by the participants’ legal guardians/next of kin.

## Author contributions

NA: writing—original draft preparation. NA, FB, SÜ, EHK, ÖMÇ, PU, ÖÖ, SO, AA, AVW, AV, and MÇ: writing—review and editing, data curation, investigation, and methodology. NA, FB, AA, and AW: formal analysis, visualization, and software. MÇ, SO, and ÖÖ: conceptualization. MÇ and AV: resources, supervision, and funding acquisition. MÇ: project administration. All authors contributed to the article and approved the submitted version.
