# Supplementary material for: Changes in the superior longitudinal fasciculus and anterior thalamic radiation in the left brain are associated with developmental dyscalculia
Source: Front Hum Neurosci. 2023 Sep 28;17:1147352. doi: 10.3389/fnhum.2023.1147352 (PMC10586317; doi:10.3389/fnhum.2023.1147352)
Supplement: Supplementary file 1 [file Data_Sheet_1.pdf]

Frontiers in Human Neuroscience

SUPPLEMENTARY MATERIAL

Changes in the superior longitudinal fasciculus and anterior thalamic radiation in the left brain are associated with developmental dyscalculia

Nazife Ayyıldız[a,b,*], Frauke Beyer, Sertaç Üstün, Emre H. Kale, Öykü Mançe Çalışır, Pınar Uran, Özgür Öner, Sinan Olkun, Alfred Anwander, A. Veronica Witte, Arno Villringer, Metehan Çiçek

*** Correspondence:** Nazife Ayyıldız: ayyildiz@cbs.mpg.de

[a] Department of Neurology, Max Planck Institute for Cognitive and Brain Sciences, Stephanstrasse 1a, 04103 Leipzig, Germany

[b] Department of Interdisciplinary Neuroscience, Health Sciences Institute & Brain Research Center, Ankara University, Ankara, Türkiye

Supplementary Tables and Notes

Table S1. Eddy quality assessment parameters before and after parameter adaptation and exclusion of the bad data.

	**QA* values of the whole sample (N=12/DD: N=16/Control)**		**QA values after exclusion of two data sets and changing outlier SD (N=10/DD: N=16/Control)**	
**Parameters**	Minimum	Maximum	Minimum	Maximum
Signal-to-Noise (SNR) (for b0 images)	13.11	25.88	13.11	25.88
Contrast-to-Noise (CNR) (for DWI images)	**0.42**	1.06	**0.58**	1.06

Absolute Motion (mm)	0.18	**4.18**	0.18	**1.91**
Relative Motion (mm)	0.08	**2.28**	0.08	**1.04**
Total Outliers Percentage (%)	0	**4.89**	0	**2.33**
(Slice-to-Volume) Translation (x, y, z) (mm)	(0) 0	**(<1.5) <3**	(0) 0	**(0.7) <1.5**
Rotation (x y, z) (degree)	(0) 0	**(<1.5) <2**	(0) 0	**(0.5) <1.5**
Eddy Current Linear Terms	0	**0.25**	0	**0.04**

*: Quality Assurance, FSL Eddy QUAD and SQUAD parameter results

Table S1 (Note S1). As a conclusion for the processes of the pre-processing steps, we finally included 10 DD and 16 TD children's DTI data. We used the default parameter for the detection of outliers defined by the slice intensity being 4 standard deviations (SDs) below the mean for outlier detection and replacement parameter except for one subject whose SD threshold was increased to 5 to keep all slices without visible artefacts. Thus, maximum total outlier percentage in the data was 2.33 % in all slices in all volumes for any subject. Minimum CNR value was 0.58 and maximum absolute and relative motion measures were 1.91mm and 1.04mm, respectively. The maximum relative motion in the data was less than one voxel size (i.e., 1.55x1.55x1.55 mm^3).

Table S1 (Note S2). Despite no significant differences between groups before excluding the two outlying subjects [for absolute motions t (26) = -1.37, p= 0. 141, means±SD 0.7±0.45mm for TD and 1.36±1.41mm for DD; for relative motions t (26) = -1.57, p= 0. 195, means±SD 0.23±0.23mm for TD and 0.49±0.64mm for DD], after excluding the two outlying subjects the t-scores and variance differences between DD and TD decreased considerably [for absolute motions t (24) = -0.49, p= 0.634, means±SD 0.7±0.45mm for TD and 0.81±0.63mm for DD; for relative motions t (24) = -0.23, p= 0.822, means±SD 0.23±0.23mm for TD and 0.24±0.15mm for DD].

Note S3: There were no significant group differences in the right SLF-temporal, right CST, right ATR, bilateral SLF-parietal, bilateral ILF and forceps major tracts in terms of tract length values (mm). The ANCOVA results and descriptive statistics can be seen below. TRACULA software calculates the tract length values as voxel numbers by default, thus we transformed the voxel numbers to the standard unit "mm" by multiplying them by 1.55mm, as one voxel dimension is 1.55mm in our study.

Actual mean length values (mm) of the groups (i.e., without correcting for eTIV):

[Mean±SD 80.7mm±5.4 for TD and 75.2mm±4 for DD for the left ATR

Mean±SD 93.6mm±5.8 for TD and 86mm±7.5 for DD for the left SLF-temporal/AF

The left SLF-parietal tract: [F (1,23) = 1.88, p = 0.18, partial $\eta^2 = 0.08$, EstimatedMean ± StandardError (SE) = 67.09mm±1.42 for TD and EstimatedMean ± SE = 70.28mm±1.81], and [mean±SD 67.62mm±6.12 for TD and 69.44mm±6.28 for DD].

The right SLF-parietal tract: [F (1,23) = 0.2, p = 0.66, partial $\eta^2 = 0.008$], EstimatedMean ± SE = 70.35mm±1.51 for TD and EstimatedMean ± SE = 69.25mm±1.93], and [mean±SD 70.72mm±6.55 for TD and 68.67mm±5.52 for DD].

The right SLF-temporal: [F (1,23) = 0.38, p = 0.54, partial $\eta^2 = 0.016$], EstimatedMean ± SE = 89.3mm±1.67 for TD and EstimatedMean ± SE = 87.6mm±2.12], and [mean±SD 89.8mm±7.21 for TD and 86.8mm±6.62 for DD].

The right CST: [F (1,23) = 2.13, p = 0.16, partial $\eta^2 = 0.085$], EstimatedMean ± SE = 111.36mm±2.33 for TD and EstimatedMean ± SE = 105.78mm±2.97], and [mean±SD 111.99mm±11.34 for TD and 104.78mm±5.72 for DD].

The right ATR: [F (1,23) = 0.74, p = 0.4, partial $\eta^2 = 0.031$], EstimatedMean ± SE = 78.28mm±0.99 for TD and EstimatedMean ± SE = 76.88mm±1.26], and [mean±SD 79.05mm±5.34 for TD and 75.64mm±6.15 for DD].

The left ILF: [F (1,23) = 2.7, p = 0.11, partial $\eta^2 = 0.11$], EstimatedMean ± SE = 95.82mm±2.55 for TD and EstimatedMean ± SE = 88.95mm±3.25], and [mean±SD 96.39mm±11.42 for TD and 88mm±8.2 for DD].

The right ILF: [F (1,23) = 0.69, p = 0.42, partial $\eta^2 = 0.03$], EstimatedMean ± SE = 96.12mm±2.67 for TD and EstimatedMean ± SE = 92.5mm±3.4], and [mean±SD 97.07mm±12.59 for TD and 90.99mm±9.42 for DD].

The forceps major: [F (1,23) = 0.33, p = 0.57, partial $\eta^2 = 0.01$], EstimatedMean ± SE = 140.44mm±2.76 for TD and EstimatedMean ± SE = 137.85mm±3.52], and [mean±SD 142.21mm±16.61 for TD and 135.01mm±9.34 for DD].

Note S4. Minimum and maximum tract length values of the groups (mm):

The left SLF-temporal/AF tract length values of the TD group (mm):

Minimum length range= [54.25mm-71.3mm], mean±SD=59.87mm±4.42 and maximum length range= [131.75mm-189.1mm], mean±SD=158.1mm±16.15.

The left SLF/AF tract length values of the DD group (mm):

Minimum length range= [51.15mm-60.45mm], mean±SD=56.89mm±3.19 and maximum length range= [128.65mm-155mm], mean±SD=146mm±8.61.

ANCOVA results:

No significant group differences for the minimum and maximum lengths of the left SLF/AF tract controlling the eTIV, respectively, $F(1,23) = 1.84$, $p = 0.188$, partial $\eta^2 = 0.074$ and $F(1,23) = 2.98$, $p = 0.098$, partial $\eta^2 = 1.12$.

Estimated mean difference ± SE = 1.94mm ± 1.43 for the minimum tract length (corrected for eTIV)

Estimated mean difference ± SE = 8.74mm ± 5.07 for the maximum tract length (corrected for eTIV)

The left ATR tract length values of the TD group (mm):

Minimum length range= [40.3mm-58.9mm], mean±SD=47.76mm±4.74 and maximum length range= [119.35mm-184.45mm], mean±SD=145.41mm±20.43.

The left ATR tract length values of the DD group (mm):

Minimum length range= [35.65mm-51.15mm], mean±SD=45.88mm±4.8 and maximum length range= [119.35mm-167.4mm], mean±SD=142.91mm±15.55.

ANCOVA results:

No significant group differences for the minimum and maximum lengths of the left ATR tract controlling the eTIV, respectively, $F(1,23) = 0.35$, $p = 0.554$, partial $\eta^2 = 0.015$ and $F(1,23) = 0.006$, $p = 0.94$, partial $\eta^2 = 0.00025$.

Estimated mean difference ± SE = 1.15mm ± 1.92 for the minimum tract length (corrected for eTIV)

Estimated mean difference ± SE = 0.57mm ± 7.48 for the maximum tract length (corrected for eTIV)

Table S2. Center of mass/ gravity (centroid) coordinates of the endpoints for each group and the distances between the mass coordinates

	Left frontal endpoint coordinates for the left SLF-temporal/AF (see Fig S4 top)			Left temporal endpoint coordinates for the left SLF-temporal/AF (see Fig S4 bottom)		
	SLF_E1x	SLF_E1y	SLF_E1z	SLF_E2x	SLF_E2y	SLF_E2z
TD	-49,054459	3,758915	21,261965	-52,091677	-38,941276	-7,132657
DD	-49,230077	4,439693	19,596931	-52,156879	-37,212847	-8,221539

distance_mm (TD – DD)	0,175618	-0,680778	1,665034	0,065202	-1,728429	1,088882
	Left prefrontal endpoint coordinates for the left ATR (see Fig S5 top)			Left anterior thalamus endpoint coordinates for the left ATR (see Fig S5 bottom)		
	ATR_E1x	ATR_E1y	ATR_E1z	ATR_E2x	ATR_E2y	ATR_E2z
TD	-25,152857	47,904429	4,976526	-6,80941	-13,832096	6,016664
DD	-25,90436	47,504698	8,343925	-6,813393	-13,795905	5,934314
distance_mm (TD – DD)	0,751503	0,399731	-3,367399	0,003983	-0,036191	0,08235

Note S5. Each yellow cell Table S2 shows that the distances (unit "mm") of the "TD – DD" between the endpoints for each tract

For the SLF:

~Distance between the frontal endpoints: +x 0.2, -y 0.7 and +z 1.7; interpretation: The frontal endpoints of the TD group seem more dorsal i.e., more superior-posterior (a little more medial as well).

~Distance between the temporal endpoints: +x 0.07, -y 1.7, and +z 1.1; interpretation: The temporal endpoints of the TD group seem more dorsal i.e., more superior-posterior.

For the ATR:

~Distance between the prefrontal endpoints: +x 0.8, +y 0.4 and -z 3.4; interpretation: The prefrontal endpoints of the TD group seem more ventral i.e., more ventral i.e. more inferior-medial (a little more anterior as well).

~Distance between the anterior thalamus endpoints: +x 0.004, -y 0.04, and +z 0.08; interpretation: The anterior thalamus endpoints of the TD group seem to be similar to the DD group.

Meaning of the axes/ directions in the MNI coordinates: -x left (lateral/medial), +x right (lateral/medial), -y posterior, +y anterior, -z inferior/ ventral, +z superior/ dorsal. Voxel-size: 1.55mm x 1.55mm x 1.55mm.

Supplementary Figures

Fig S1

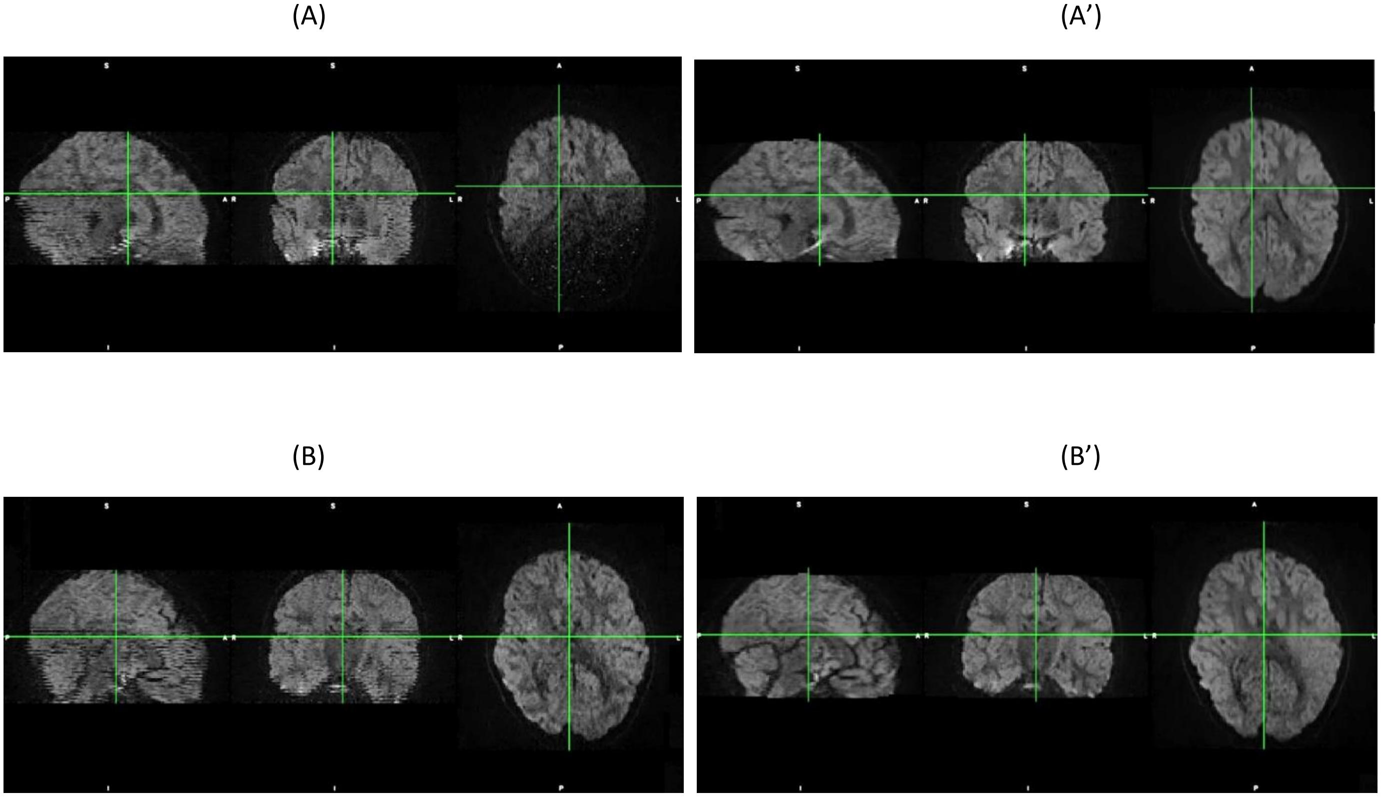

Fig S1 (A and B) show images with slices showing a signal drop-out (partly black slice in A) and zig-zag patterns occurred (A and especially in B) because of excessive movements between slices and within volumes. After eddy-correction with outlier replacement and slice-to-volume corrections, the signal was reconstructed resulting in no signal loss in A’ and no zig-zag patterns in A’ and B’.

Fig S2

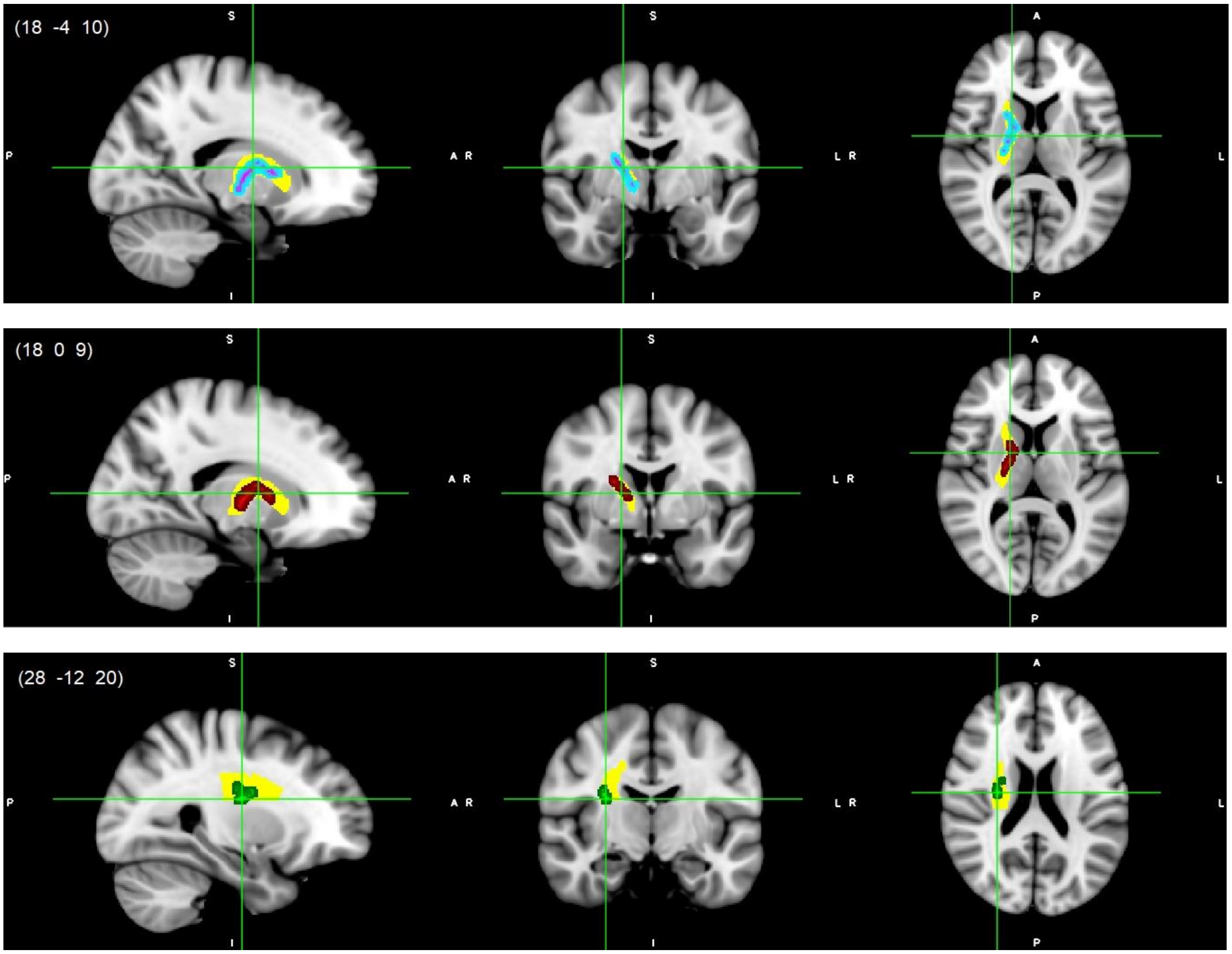

Fig S2 Right IC (top/middle) and right SCR (bottom) white matter microstructure differences. Higher AD (light blue-pink, top), higher MD (red, middle) and lower FA (green, bottom) values in DD compared to normally developing children, $p<0.05$, FWE corrected with TFCE. Results are thickened with tbss_fill. Yellow regions represent selected IC and SCR masks as ROIs. IC, Internal Capsule; SCR, Superior Corona Radiate.

Fig S3

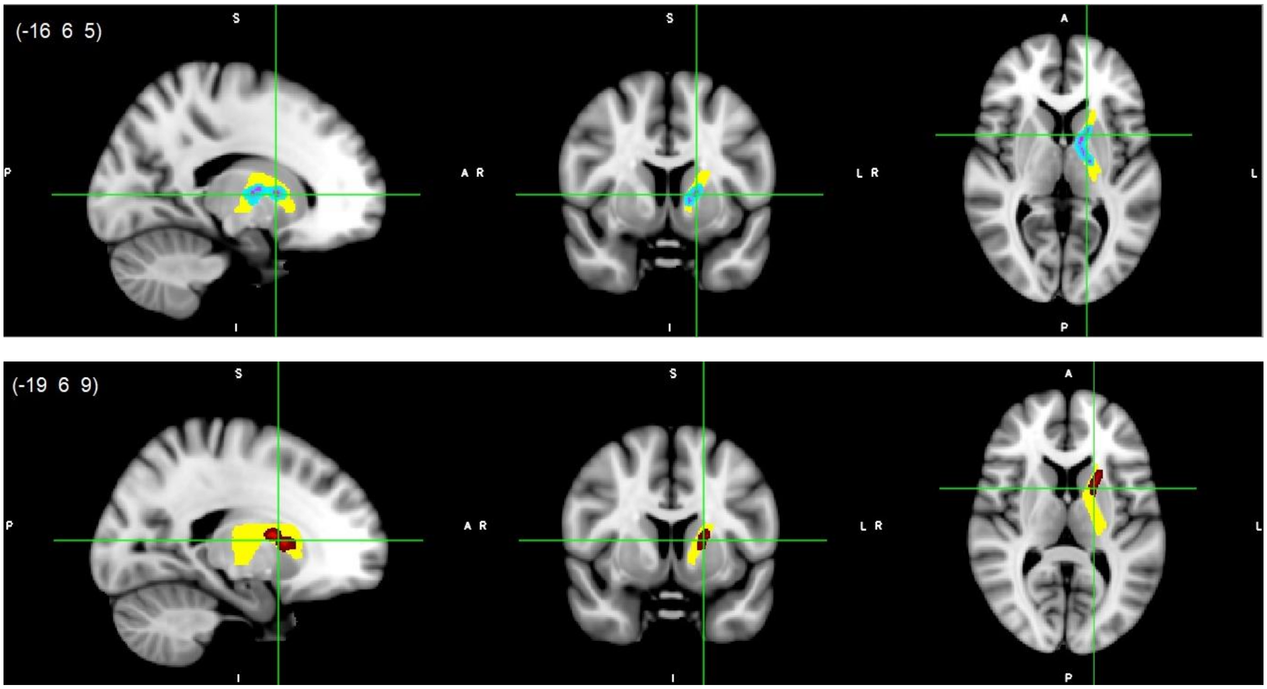

Fig S3 Left IC white matter microstructure differences. Higher AD (light blue-pink, top) and higher MD (red, bottom) values in DD compared to normally developing children, $p<0.05$, FWE corrected with TFCE. Results are thickened with tbss_fill. Yellow region represents selected IC mask as ROI. IC, Internal Capsule.

Fig S4

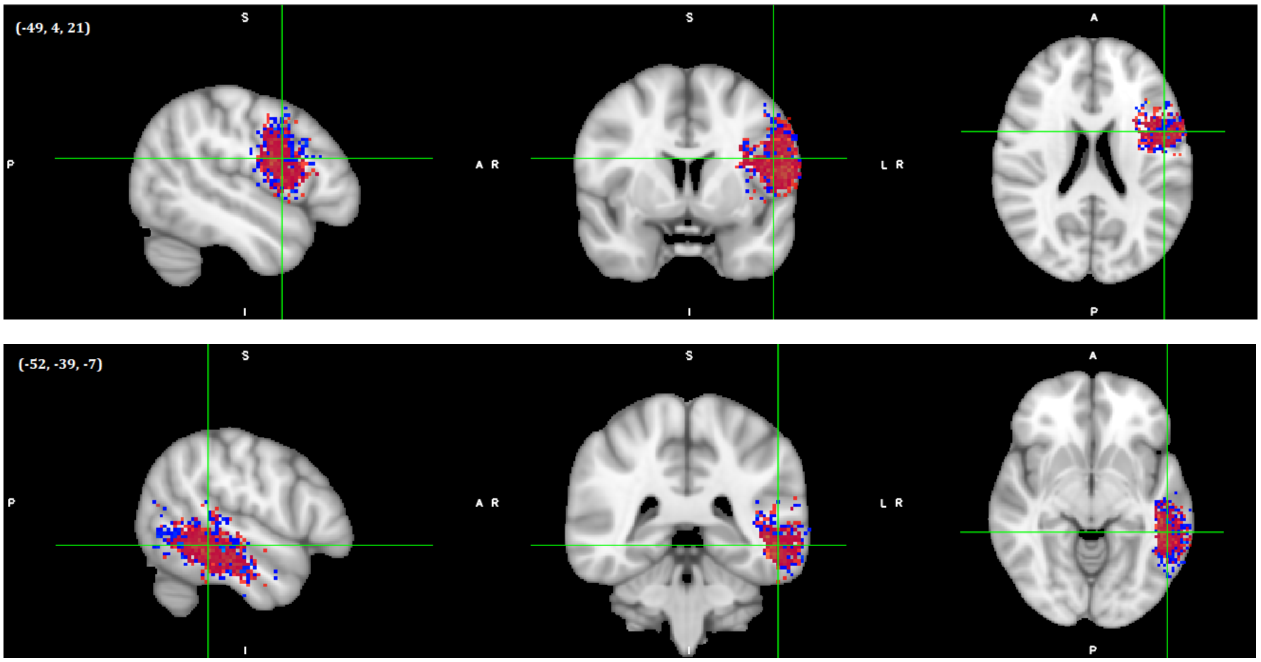

Fig S4 Endpoints for the left SLF-temporal/AF pathway reconstruction in global tractography analyses. Frontal endpoint (top) and temporal endpoint (bottom). Red colors represent DD and blue colors represent TD samples. Images shown here are on MNI152 template. Center of gravity coordinates of the frontal endpoints were (-49.1, 3.8, 21.3) and (-49.2, 4.4, 19.6) for TD and DD groups, respectively. Center of gravity coordinates of the temporal endpoints were (-52.1, -38.9, -7.1) and (-52.2, -37.2, -8.2) for TD and DD groups, respectively.

Fig S5

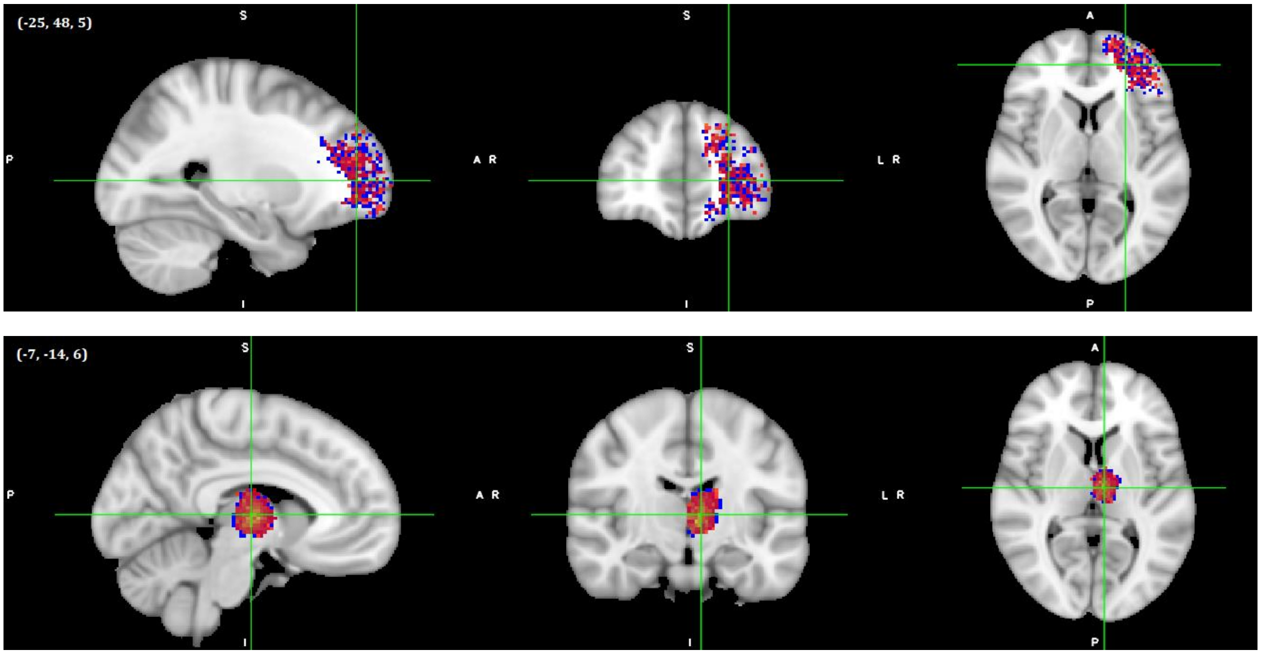

Fig S5 Endpoints for the left ATR pathway reconstruction in global tractography analyses. Prefrontal endpoint (top) and anterior thalamus endpoint (bottom). Red colors represent DD and blue colors represent TD samples. Images shown here are on MNI152 template. Center of gravity coordinates of the prefrontal endpoints were (-25.2, 47.9, 5) and (-25.9, 47.5, 8.3) for TD and DD groups, respectively. Center of gravity coordinates of the thalamus endpoints were (-6.8, -13.8, 6) and (-6.8, -13.8, 5.9) for TD and DD groups, respectively.
